# Using ROS as a Second Messenger, NADPH Oxidase 2 Mediates Macrophage Senescence via Interaction with NF-*κ*B during *Pseudomonas aeruginosa* Infection

**DOI:** 10.1155/2018/9741838

**Published:** 2018-06-27

**Authors:** Hui Li, Yi-Feng Luo, Yong-Sheng Wang, Qing Yang, Yong-Long Xiao, Hou-Rong Cai, Can-Mao Xie

**Affiliations:** ^1^Department of Respiratory Medicine, the Affiliated Drum Tower Hospital, Nanjing University Medical School, Nanjing 210008, China; ^2^Department of Respiratory Medicine, the First Affiliated Hospital of Sun Yat-sen University, Guangzhou, China; ^3^Department of Respiratory Medicine, the Second Affiliated Hospital of Nanchang University, Nanchang, China

## Abstract

*Pseudomonas aeruginosa* (PA) is one of the most prevalent pathogens that cause nosocomial infection in critical patients. However, the mechanisms underlying macrophage growth status and functional changes during PA infection are yet unknown. In the present study, NADPH oxidase, gp91^phox^ (NOX2) mediated macrophage to senescence in a PAO1 colony-dependent manner. gp91^phox^ might regulate the senescence process through mutual interaction with the NF-*κ*B pathway. During infection, the overexpression or downregulation of gp91^phox^ in macrophage could affect the nuclear activity of NF-*κ*B p65, while the downregulation of NF-*κ*B p65 led to a suppressed expression of gp91^phox^. Reactive oxygen species (ROS) served as the second messenger between both molecules as the ROS inhibitor, *N*-acetylcysteine (NAC), could partially restore these changes. Consequently, the level of ROS and inflammatory cytokines, including IL-6 and TNF*α*, elevated during PAO1 infection, and their production altered as a result of the genetic manipulation of gp91^phox^ and NF-*κ*B p65, as well as NAC treatment. Also, the senescent phenotypes, SA-*β*-gal staining and p16^ink4a^, changed after genetic manipulation with gp91^phox^ and NF-*κ*B p65 and NAC treatment. The capacity of phagocytosis in macrophages was decreased during senescence. In conclusion, PA directs the macrophage towards senescence, and senescent macrophages exhibit a decreased ability of phagocytosis. This process of senescence was regulated by the interactions between NADPH oxidase gp91^phox^ and NF-*κ*B p65 via ROS as a second messenger.

## 1. Introduction

*Pseudomonas aeruginosa* (PA) is one of the most prevalent pathogens that cause nosocomial infection, especially in critically ill patients, resulting in a high rate of mortality [1–3]. In the lung, PA possesses a sophisticated toxin secretion system that can damage the epithelial integrity, causing bacteremia [4]. The host response to PA is complex and involves multiple cell types along with the activation of a variety of genes. During the early stages of infection, the host primarily relies on the innate immune cells including neutrophils and macrophages to combat PA infection. For example, macrophage recognizes and phagocytoses the pathogens and secretes the proinflammatory cytokines, such as tumor necrosis growth factor-alpha (TNF*α*) and interleukin- (IL-) 6 that displayed an effective defense mechanism against bacterial and viral pathogens [5]. Inflammatory cytokine release is one of the characteristics of bacterial infections; however, exaggerated inflammations cause tissue damage, thereby leading to high morbidity and mortality. The mortality after PA infection was found to be related to bacterial load in the lung [6]. Thus, the infection with high bacterial load is speculated to lead to deteriorated inflammation niches. However, the effect of exaggerated inflammation on inflammatory cells, such as the growth status of macrophages, is not yet fully understood.

During infection, the reactive oxygen species (ROS) that are involved in abolishing foreign insults are mainly generated by nicotinamide adenine dinucleotide phosphate (NADPH) oxidase in phagocytes. Furthermore, ROS continue to participate in the complex physiological processes including cell signaling and proliferation and play a critical role in disease pathogenesis [7, 8]. NADPH oxidases (Noxs), such as NOX1–5 and DUOX1–2, are membrane-associated enzymes using NADPH as an electron donor to catalyze the reduced form of molecular oxygen to superoxide and hydrogen peroxide (H_2_O_2_) [9]. Of these, the multicomponent enzyme NOX2 is the major source of ROS production and is widely distributed on the phagocytes including neutrophils and monocytes/macrophages in the lungs [10]. The NOX2 complex is an assembly of five proteins, including the membrane proteins gp91^phox^/NOX2 and p22^phox^ that constitute the core of the complex. The catalytic site and the NADPH-binding site are contained within the gp91^phox^/NOX2 protein. The smaller membrane protein, p22^phox^, might function as a stabilizing factor of the gp91^phox^/NOX2 protein complex, as well as an attachment site for the cytoplasmic subunit p47^phox^ [11]. During infection, NOX2 catalyzes the abundant production of ROS to abolish the bacteria in the phagocytes. As described above, this infection-intrigued oxidative stress might also regulate the growth status of the phagocytes.

Nuclear factor-*κ*B (NF-*κ*B) is an inflammatory transcription factor. During the bacterial infection in the cytoplasm, the heterodimer of NF-*κ*B p65 and p50 is uncoupled from I*κ*B*α* and translocated to the nucleus, wherein p65 binds upstream of the inflammatory cytokines and regulates their expression [12]. As reported previously, NOX2 served as an exaggerator or an inhibitor of inflammation by regulating NF-*κ*B activity [13, 14]. The depletion of NOX2 leads to neutrophil pyroptosis and causes excessive lung inflammation during PA infection [15]. Hence, a regulatory loop consisting of NOXs and NF-*κ*B that coordinate the oxidative and inflammatory reaction in phagocytes can be speculated; however, this regulatory mechanism might engender specific cellular niches affecting the cell fate. Cellular senescence is a status of cell growth arrest, which is affected by inflammation and oxidative stress. Hence, we deduced that the regulation between NOX2 and NF-*κ*B could determine the growth status of macrophage, dependent on the severity of infection (bacteria loads), thereby affecting the host defense.

## 2. Materials and Methods

### 2.1. Preparation of *P. aeruginosa* Strain PAO1

Initially, PAO1 colonies were cultured on a Luria-Bertani (LB) agar plate at 37°C overnight, followed by inoculation into 10 mL fresh LB medium for a continued overnight growth. After another inoculation of PAO1 to fresh LB broth, this medium was cultured to reach an optimal density that was used for the infection model at various colony-forming units (CFUs).

### 2.2. Preparation of Macrophage

RAW264.7 cells were inoculated in a 6-well plate with RPMI 1640 medium at a density of 5 × 10^5^/L overnight at 37°C. For the infectious macrophage model, PAO1 was seeded in a 6-well plate at a density of 4 × 10^6^/well and 1.6 × 10^7^/well, respectively. Subsequently, the cells were harvested at 4 and 24 h, respectively. For ROS inhibition assay, the ROS inhibitor, NAC (stock concentration: 100 mM, working concentration: 1 mM; Sigma-Aldrich Co., St Louis, MO, USA), was added 30 min before PAO1 infection and continued until the cells were harvested.

### 2.3. Detection of Cellular Senescence

SA-*β*-gal staining was performed on cultured macrophages according to the manufacturer's instructions (Beyotime Biotech, China). Furthermore, p16^ink4a^ antibody (Abcam, Shanghai, China) was used for the detection of cellular senescence.

### 2.4. Lentivirus Infection and Gene Manipulations

To investigate the interaction between NOX2 and NF-*κ*B, gp91^phox^ was knocked down and overexpressed using a lentivirus infection system with plasmid pLVX-shRNA2 (Clontech Co., USA); also, the *p65* gene was knocked down. Briefly, the cells were transfected with a Lenti-X™ lentiviral expression system using the kit for lentiviral packaging (Yuduo Biotech, Shanghai, China) according to the manufacturer's protocol. 5 × 10^5^ macrophages were plated in 25 cm^2^ cell culture flasks. After 18 h, polybrene was added at the final concentration of 8 mg/mL, and 100 mL virus supernatant was added, followed by continued culture for 24 h. Subsequently, the RPMI-1640 medium containing lentivirus was replaced with a normal RPMI-1640 complete medium. Next, 2 mg/mL puromycin was added every 2 days for screening for a total of 7 days, after which the cell lines were stably infected with control, pLVX-gp91^phox^KDshRNA2, pLVX-gp91^phox^OEshRNA2, and pLVX-p65KDshRNA2 plasmids, respectively. Finally, macrophages with different gene manipulations were cultured and treated at the appropriate time points.

### 2.5. Western Blotting

Primary anti-NF-*κ*B p65, gp91^phox^, Na-K-ATPase, *β*-actin, and histone H3 antibodies were purchased from Santa Cruz Biotechnology (Santa Cruz, CA, USA). Phospho-NF-*κ*B p65 (Ser536) was obtained from Cell Signaling Technology (Danvers, MA, USA). Western blotting was performed on the cell, membrane protein, and nuclear extracts as described previously [16]. *β*-Actin was used as the control for whole cell extract proteins, histone H3 for nuclear proteins, and Na-K-ATPase for the membrane proteins.

### 2.6. ROS Production Detection

Cells were harvested at the indicated time points and ROS production determined following the manufacturer's instructions using a commercial kit (Huijia Biotech, China). The results of the assay were measured on a BioTek ELISA plate reader (BioTek Instruments, Inc., VT, USA) at 405 nm.

### 2.7. Proinflammatory Cytokine Expression

Cells were harvested at the indicated time points. Real-time PCR was used to detect the expression level of *TNFα*, *IL-10*, and *IL-6* genes using the following primers: *tnfα* (forward: 5′-AGGATAACTGGAACACAGACA-3′; reverse: 5′-TTGGAGACAACATACAAGCA-3′), *il-10* (forward: 5′-CATACTGCTAACCGACTCCT-3′; reverse: 5′-AATGCTCCTTGATTTCTGG-3′), *il-6* (forward: 5′-GCCTTCCCTACTTCACAA-3′; reverse: 5′-ACAACTCTTTTCTCATTTCCAC-3′), and *gapdh* (forward: 5′-GCAGTAAACAGTCCATCTACAA-3′; reverse: 5′-CTCTCCTTCATCCACCCT-3′). The relative expressions were calculated as 2^−ΔΔCT^.

### 2.8. Phagocytosis Assay

Wild-type (WT) RAW264.7 macrophages and those with different genetic manipulations were seeded in a 6-well plate at a density of 5 × 10^5^ cells/well overnight. Then, the cells were treated with PAO1 at colony counts of 4 × 10^6^/well and 1.6 × 10^7^/well for 4 h followed by treatment with fluorescently labeled latex beads at a concentration of 10 × 10^7^ beads/mL (Invitrogen) for an additional 4 h. The unstimulated WT macrophages with or without empty vector were used as control. NAC treatment was conducted for 30 min before PAO1 infection in pLVX-gp91^phox^OEshRNA2 macrophage to elucidate whether ROS inhibition could affect the macrophage phagocytosis. Then, the cells were washed three times with phosphate-buffered saline (PBS) to remove the unused beads. Extracellular fluorescence was quenched by Trypan Blue, and the phagocytosis of the fluorescent-labeled beads by RAW264.7 was measured using a FLUOstar Optima fluorimeter (BMG LABTECH Co., Ortenberg, Germany) at an excitation wavelength of 480 nm and emission wavelength 520 nm. Data were expressed as relative fluorescent units (RFUs).

### 2.9. Statistical Analysis

To evaluate the differences between the groups, analyses were performed using GraphPad Prism 5.0 software by an unpaired 𝑡-test (two groups) or ANOVA (multiple groups) followed by the Tukey-Kramer multiple comparison post-test. Nonparametric ANOVA with Kruskal-Wallis was used for the post hoc test. The results were represented as mean ± SEM. Two-tailed *p* values < 0.05 were considered significant.

## 3. Results

### 3.1. NADPH Oxidase Activation, Oxidative Stress, and NF-*κ*B Activation Are Involved in Macrophage Senescence during PA Infection

**Figure 1:**
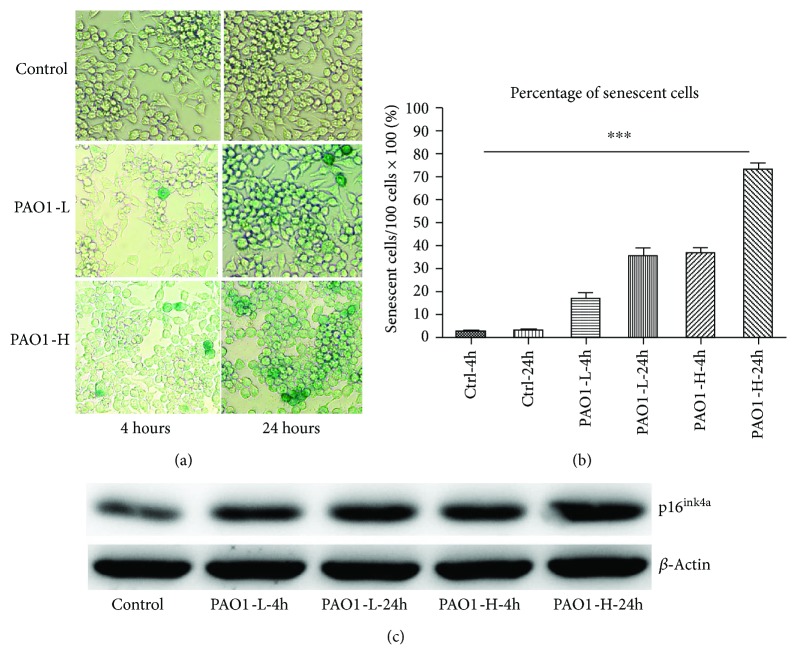
PAO1 infection causes macrophage senescence in a colony-dependent manner. (a) Macrophages underwent senescence indicated by positive SA-*β*-gal staining during the PAO1 infection. (b) Percentage of senescent cells in macrophages incubated with the indicated colony counts of PAO1 for 4 and 24 h. ^∗∗∗^*p* < 0.0001 by ANOVA test among these groups. The percentage was calculated as the mean value of the percentage of senescent cells under 5 random fields of a high-power microscope. Three independent experiments were conducted. (c) Senescent marker, p16^ink4a^, expression among the different groups. Control indicates untreated macrophages incubated under standard condition for 24 h; PAO1-L-4h indicates that the macrophages were incubated with 4 × 10^6^ colony counts of PAO1 for 4 h; PAO1-L-24h indicates that the macrophages were incubated with 4 × 10^6^ colony counts of PAO1 for 24 h; PAO1-H-4h indicates that the macrophages were incubated with 1.6 × 10^7^ colony counts of PAO1 for 4 h; PAO1-H-24h indicates that the macrophages were incubated with 1.6 × 10^7^ colony counts of PAO1 for 24 h.

PAO1 at colony counts of 4 × 10^6^ CFU and 1.6 × 10^7^ CFU was incubated with macrophages RAW264.7 for 4 and 24 h, respectively. Consequently, PA caused macrophage senescence in a time- and colony count-dependent manner. As shown in Figures 1(a) and 1(b), PAO1 at 4 × 10^6^ CFU resulted in approximately 15% cellular senescence at 4 h coincubation with macrophages, which was increased to 35% at 24 h. PAO1 at 1.6 × 10^7^ CFU further increased the percentage of cellular senescence, and after coincubation with PAO1 at 1.6 × 10^7^ CFU for 24 h, more than 70% macrophages exhibited positive SA-*β*-gal staining. Furthermore, to confirm the cellular senescence occurring after PAO1 infection, the expression of a senescence-associated biomarker, p16^ink4a^, was detected. As shown in Figure 1(c), the expression of p16^ink4a^ was greatly increased in macrophages after PAO1 infection in a time- and colony count-dependent manner.

**Figure 2:**
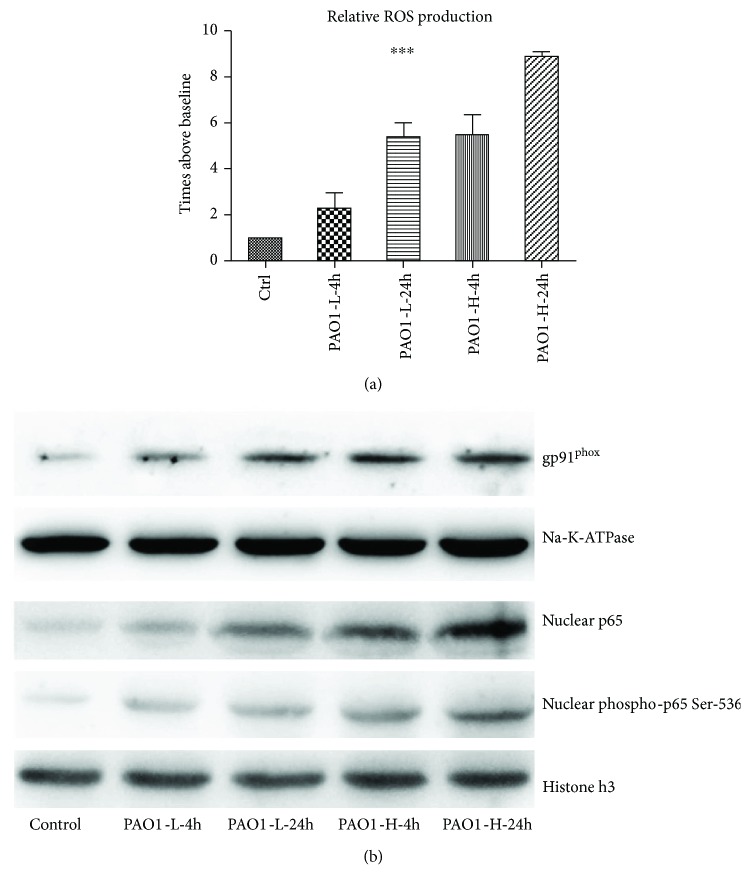
PAO1 infection in macrophages intrigues ROS production and the activation of NADPH oxidase elements gp91^phox^ and nuclear NF-*κ*B p65. (a) ROS production during PAO1 infection in macrophages was time- and colony count-dependent; ANOVA test showed a significant difference among these groups (*p* < 0.0001). Three independent experiments were conducted; (b) after PAO1 infection, an increased expression of gp91^phox^ was observed, following a prolonged duration and increased colony count infection (upper line; Na-K-ATPase was used as control); nuclear NF-*κ*B p65 expression and phosphorylation at Ser536 were elevated during PAO1 infection in macrophage (lower line; histone H3 was used as control). ^∗∗∗^*p* < 0.0001 using ANOVA test for these groups.

Because cellular senescence is highly related to oxidative stress, next, we determined the ROS production to investigate the oxidative status in macrophages during PAO1 infection. After 24 h, the macrophages treated with 1.6 × 10^7^ CFU PAO1 produced > 8-fold ROS as compared to the control cells treated with vehicle at a time- and colony count-dependent manner (Figure 2(a)). Subsequently, we investigated the expression of a ROS generator, NADPH oxidase element, gp91^phox^.  Figure 2(b) reveals an increased expression of gp91^phox^ after PAO1 treatment in macrophages. Taken together, NADPH oxidase elicited the ROS production during PAO1-induced macrophage senescence.

According to previous reports [17], NF-*κ*B activation is usually accompanied by oxidative stress during infection. As indicated in Figure 2(b), the expression of nuclear NF-*κ*B p65 was increased in RAW264.7 cells after 4 and 24 h of PAO1 infection, suggesting that the nuclear translocation of p65 increased during this process. Furthermore, the high activity of NF-*κ*B p65 was shown as increased phosphorylation of p65 at Ser536 through the infection (Figure 2(b)). These results suggested that nuclear NF-*κ*B p65 was activated in senescent macrophages during PAO1 infection.

### 3.2. NADPH Oxidase Subunit gp91^phox^ Regulates p65 Activation via ROS as the Second Messenger, Leading to Macrophage Senescence during PAO1 Infection

**Figure 3:**
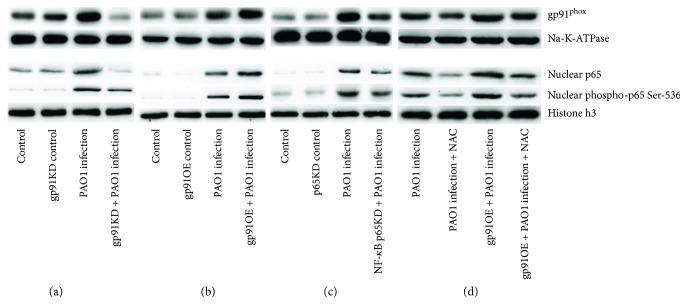
Mutual interaction between gp91^phox^ and NF-*κ*B p65 during PAO1 infection in RAW264.7 macrophages. (a) After 24 h, the macrophages were incubated with PAO1 at 1.6 × 10^7^ CFU, and the knockdown of gp91^phox^ expression caused a decreased nuclear NF-*κ*B p65 and Ser536 phosphorylation. (b) The overexpression of gp91^phox^ led to increased nuclear NF-*κ*B p65 and its phosphorylation at Ser536. (c) The downregulated expression of NF-*κ*B p65 affected gp91^phox^ expression. (d) ROS scavenger, NAC, effectively reversed the level of gp91^phox^ and nuclear p65 induced in PAO1 infection and PAO1-infected gp91^phox^-overexpressed macrophages. All experiments were set up at 24 h post PAO1 infection. Note: NAC concentration: 1 mM.

Since both gp91^phox^ and NF-*κ*B p65 were involved in PAO1-induced macrophage senescence, we postulated an underlying interaction mechanism. Next, gp91^phox^ (termed as pLVX-gp91phoxOEshRNA2 and pLVX-gp91phoxKDshRNA2, resp.) was upregulated and downregulated through genetic manipulation using a lentivirus system, and NF-*κ*B p65 was also downregulated (pLVX-p65KDshRNA2). As shown in Figure 3(a), compared to the PAO1-infected WT RAW264.7, macrophages with pLVX-gp91phoxKDshRNA2 responded to PAO1 infection as low gp91^phox^ expression and decreased nuclear NF-*κ*B p65 level and its phosphorylation on Ser536. Conversely, RAW264.7 with pLVX-gp91phoxOEshRNA2 exhibited a high nuclear NF-*κ*B p65 level and Ser536 phosphorylation than did WT RAW264.7. These results indicated that nuclear NF-*κ*B p65 activity might be regulated by the activation of gp91^phox^. Interestingly, the downregulation of NF-*κ*B p65 also decreased the expression of gp91^phox^ in PAO1-infected macrophages, thereby suggesting a feedback loop between the two molecules.

Since gp91^phox^ and NF-*κ*B p65 are localized in different cellular compartments, a direct mutual interaction is unlikely. Thus, we postulated that gp91^phox^ regulates the NF-*κ*B activity via ROS as a second messenger. The WT macrophages and gp91^phox^-overexpressing macrophages were treated with the ROS inhibitor, NAC, prior to PAO1 infection, in order to investigate the occurrence of an inhibitory effect on NF-*κ*B p65 activity. As shown in Figure 3(b), NAC treatment largely reduced the nuclear NF-*κ*B p65 and phosphorylation of p65 at Ser536 during PAO1 infection in both WT or gp91^phox^-overexpressing macrophages. Taken together, these results supported that ROS was a secondary messenger used as a regulatory factor in the interaction between gp91^phox^ and NF-*κ*B p65.

**Figure 4:**
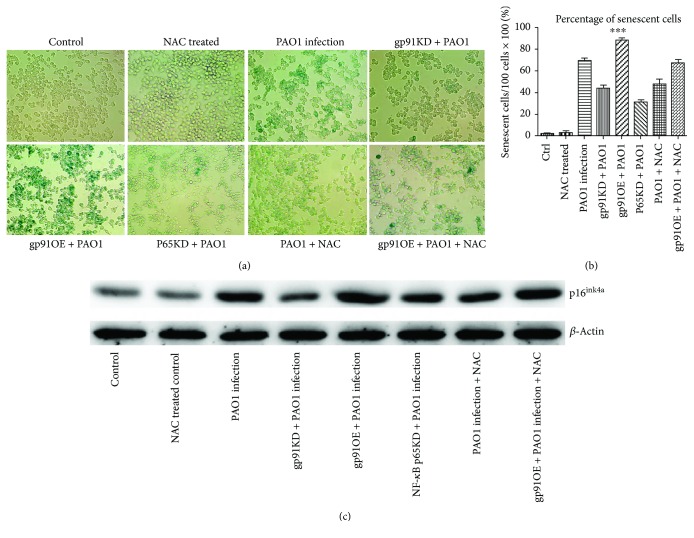
Cellular senescence determined by SA-*β*-gal staining and p16^ink4a^ among macrophages with different manipulations. (a) SA-*β*-gal staining. (b) Percentage of senescent cells; ^∗∗∗^*p* < 0.0001 ANOVA test among these groups. (c) p16^ink4a^ expression among different groups (*β*-actin was used as a control). All experiments were set up at 24 h post PAO1 infection at 1.6 × 10^7^ CFU.

Next, we examined whether this interaction loop of gp91^phox^-ROS-NF-*κ*B p65 could eventually affect the senescent phenotype of the macrophages during PAO1 infection.  Figure 4(a) revealed that during PAO1 infection, the downregulation of gp91^phox^ and NF-*κ*B p65 in macrophages decreased the cellular macrophage senescence phenotypes, including positive SA-*β*-gal staining and p16^ink4a^ expression as compared to that of the WT macrophages. Conversely, the overexpression of gp91^phox^ in macrophages resulted in an elevated proportion of cellular senescence as indicated by increased positive SA-*β*-gal staining and p16^ink4a^ expression. Moreover, compared to the PAO1-infected WT and gp91^phox^-overexpressing macrophages, the NAC treatment effectively aborted their capacity of inducing senescence in the macrophages (Figure 4(b)). Taken together, PAO1 infection induced macrophages to senescence via the regulation between gp91^phox^ and NF-*κ*B p65, mediated by ROS production as the second messenger.

### 3.3. gp91^phox^ and NF-*κ*B Coordinately Regulate the Oxidative Stress and Inflammation during PAO1 Infection-Induced Macrophage Senescence

**Figure 5:**
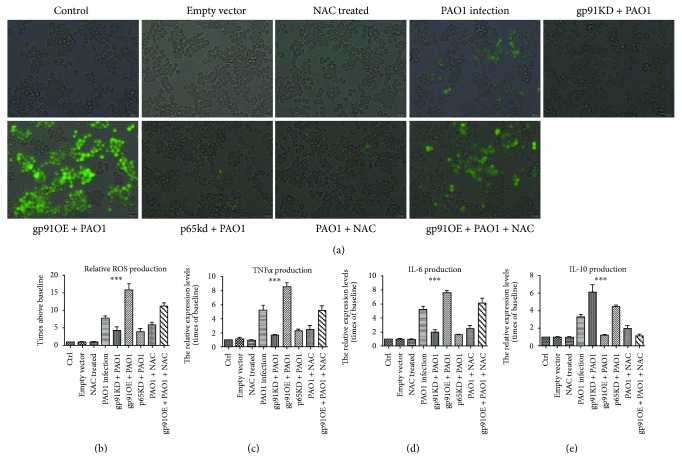
(a) Fluorescence image of ROS production. (b) Relative ROS production. (c–e) TNF*α*, IL-6, and IL-10, respectively, were determined using real-time PCR after macrophages were infected with PAO1 for 24 h. The results were summarized from three independent experiments and expressed as a relative level compared to the baseline. ^∗∗∗^*p* < 0.0001 using ANOVA test for these groups.

Since an interaction loop of gp91phox-ROS-NF-*κ*B p65 was detected in the senescent macrophages during PAO1 infection, we assessed the production of ROS and the expression of inflammatory cytokines including IL-6, IL-10, and TNF*α*. As shown in Figures 5(a) and 5(b), a significant difference was observed among these groups by one-way ANOVA test (*p* < 0.0001); compared to the control group, macrophages with PAO1 infection produced 7-fold more ROS. This elevated production was greatly inhibited in pLVX-gp91^phox^KDshRNA2 and pLVX-p65KDshRNA2 macrophages. The NAC treatment in PAO1-infected macrophages reduced ROS production by approximately 24.3% as compared to the PAO1-infected WT macrophages; however, a statistical difference in ROS production was not observed between both groups (post hoc test). During PAO1 infection, the ROS produced by pLVX-gp91^phox^OEshRNA2 macrophages was 15.7-fold above the baseline, which was significantly higher than that by WT, pLVX-gp91^phox^KDshRNA2, and pLVX-p65KDshRNA2 macrophages. NAC treatment significantly abolished the increased ROS production in pLVX-gp91^phox^OEshRNA2 macrophage that responded to the PAO1 infection.

Furthermore, to investigate the expression of inflammatory cytokines, we determined the mRNA levels of *TNFα*, *IL-6*, and *IL-10* using a real-time PCR method. As indicated in Figures 5(c)–5(e) during PAO1 infection, WT macrophage showed an increased level of *TNFα*, *IL-6*, and *IL-10* gene expression. Compared to the WT RAW264.7, pLVX-gp91phoxKDshRNA2 RAW264.7 presented reduced TNF*α* and IL-6 levels and elevated IL-10 expression (*p* < 0.05). On the other hand, pLVX-gp91^phox^OEshRNA2 macrophages displayed elevated mRNA levels of *TNFα* and IL-6 and reduced *IL-10* as compared to the pLVX-gp91phoxKDshRNA2 macrophages. In addition, pLVX-p65KDshRNA2 macrophages exhibited reduced mRNA levels of TNF*α* and IL-6 and elevated IL-10 as compared to the WT macrophage. The NAC treatment in WT or pLVX-gp91^phox^OEshRNA2 macrophages greatly decreased the IL-6 and TNF*α* expression and elevated the IL-10 expression as compared to the comparable groups. These results indicated that ROS and inflammatory cytokines were synergistically produced in senescent macrophages by mutual interactions between NOX2 and NF-*κ*B during PAO1 infection.

### 3.4. Senescent Macrophages Exhibited a Compromised Capacity of Phagocytosis

**Figure 6:**
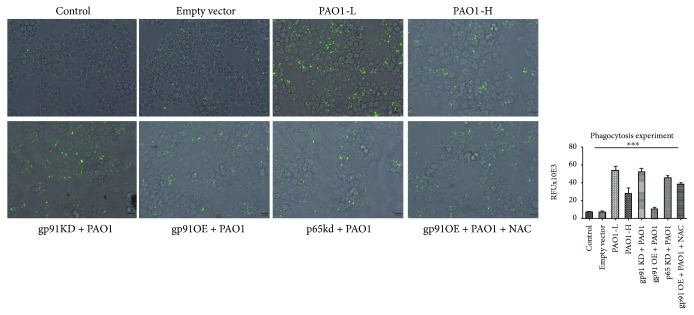
Phagocytosis assay. The phagocytosis of fluorescent-labeled beads by RAW264.7 was measured 4 h after the macrophages were infected with PAO1. Data were expressed as relative fluorescent units (RFUs). Results were summarized from three independent experiments; ^∗∗∗^*p* < 0.0001 using ANOVA test for those groups.

To evaluate the capacity of phagocytosis of senescent macrophages, that of the fluorescent-labeled beads was determined. As shown in Figure 6, after 4 h incubation with fluorescent-labeled beads, the PAO1-infected macrophages demonstrated an increased ability of phagocytosis as compared to the unstimulated macrophages without infection. Interestingly, the macrophages infected with lower bacterial load presented higher phagocytosis capacity than that infected with higher bacteria loads. Similarly, during the PAO1 infection (1.6 × 10^7^/well), the phagocytosis ability of pLVX-gp91^phox^OEshRNA2 macrophages was lower than the WT, pLVX-gp91^phox^KDshRNA2, and pLVX-p65KDshRNA2 macrophages, respectively. The NAC treatment in pLVX-gp91^phox^OEshRNA2 macrophages could reverse its decreased phagocytosis capacity under similar conditions. Also, the phagocytosis capacities among gp91^phox^KDshRNA2, pLVX-p65KDshRNA2, and NAC-treated pLVX-gp91^phox^OEshRNA2 macrophages were similar during PAO1 infection. In this study, it indicated that pLVX-gp91^phox^OEshRNA2 macrophage exhibited more sensitivity to senescence during PAO1 infection, suggesting that PA induced-senescent macrophages exhibited a compromised ability of phagocytosis.

## 4. Discussion

Herein, we reported that NADPH oxidase 2 mediated the macrophage senescence during PA infection via interaction with the NF-*κ*B pathway. Specifically, (1) PAO1 induced the macrophage senescence in a colony-dependent manner; the NOX2-dependent ROS production and the resulting oxidative stress may contribute to macrophage senescence during PAO1 infection. (2) NOX2 mutually interacted with nuclear NF-*κ*B activity to perpetuate the release of inflammatory cytokine. Of these, the ROS serve as second messengers for regulating the NF-*κ*B activation during PAO1 infection. (3) Senescent macrophages exhibit compromised phagocytosis during PAO1 infection.

Commonly, PA infection in the lungs leads to the activation of NOX2 and NOX4. As reported previously, PA infection stimulates the human lung microvascular endothelial cells (HLMECs) towards apoptosis via activation of NOX4 but not NOX2, resulting in elevated lung permeability. Moreover, the genetic downregulation of NOX2 blocks the inflammatory pathway [18]. The genetic deletion of p47^phox^ (cytosolic elements of NOX2) resulted in the inactivation of NF-*κ*B, thereby decreasing the proinflammatory secretion that impaired the bacterial clearance. In macrophages, p47^phox^ also partially affects NF-*κ*B translocation, and the whole expression of TLR4 and p47^phox^ contributes to the maximal activation of NF-*κ*B [13]. Hence, the activity of NADPH oxidase is one of the interesting factors in NF-*κ*B activation.

Although much attention is paid to the role of neutrophils during sepsis or lung infection including pyroptosis and neutrophil senescence [15, 19], little is known about the macrophage status of proliferation, senescence, or apoptosis during infection. This study indicated that during PAO1 infection, macrophages were directed towards senescence. According to previous reports [20], the activity of NADPH oxidase subunit p47^phox^ was decreased due to its decreased phosphorylation in macrophages because of p66Shc knockdown, which led to decreased ROS production that contributed to an antiaging role in long-lived p66Shc-deficient mice. Similar to neutrophil senescence [19], macrophage senescence led to enhanced inflammatory niches, which were displayed as a result of increased nuclear translocation of NF-*κ*B and proinflammatory cytokine production. The current study showed that the genetic manipulation of gp91^phox^ led to altered nuclear NF-*κ*B activity; these results were identical to previous reports [13, 18]. Oxidative stress and the inflammatory pathway have been considered to be involved synergistically in several pathogenesis mechanisms. The catalytic products of NADPH oxidase, ROS, play a significant role in cell cycle including the inhibition or perpetuation of cell growth [21, 22]. The senescence-associated secretory phenotype (SASP) controlled by NF-*κ*B is one of the characteristics of senescent cells [23]. For example, high levels of IL-1 and IL-6 can induce ROS-mediated DNA damage response (DDR) and thus contribute to cellular senescence [24]. In the current study, PAO1 infection induced macrophage senescence in an NADPH oxidase gp91^phox^ activity-dependent manner. The regulation of gp91^phox^ expression directly affected the macrophage senescence status. Consequently, the NADPH oxidase-dependent ROS production increased during PAO1 infection in the macrophages. The loss of function of gp91^phox^ led to the inactivation of the NF-*κ*B p65 subunit in the nucleus, and the overexpression of gp91^phox^ increased p65 translocation. These processes might seem unlikely to regulate both molecules as they are localized in different cellular compartments. As reported previously, ROS produced by NADPH oxidases can act as a second messenger for regulating the NF-*κ*B activation [25, 26]. ROS exerts a dual role in cell growth: cellular senescence or apoptosis or antiapoptosis-dependent NF-*κ*B activity [21, 22, 27]. Also, in this study, ROS served as a second messenger between the activity of gp91^phox^ and NF-*κ*B p65, since the ROS inhibitor, NAC, partially blocked the macrophage senescence and nuclear NF-*κ*B p65 activation. Taken together, we described a regulatory mechanism that mediated PA infection-induced macrophage senescence via interaction between NADPH oxidase gp91^phox^ and NF-*κ*B p65 using ROS as a secondary messenger.

NF-*κ*B activity exerts a protective role in preventing lymphocytes towards apoptosis through direct regulation of the expression of antiapoptotic factors, such as Bcl-xL and c-IAP2, during sepsis [28]. However, the overexpression of NF-*κ*B subunit p65 induced cell senescence through genomic stability and DNA repair [29, 30], while the loss of p50, the suppressive heterodimer of p65, led to the accelerated prematurity of the cells [31]. This phenomenon suggested that NF-*κ*B was involved in arresting cell growth in a multifaceted way. Recently, NF-*κ*B and its related inflammatory cytokines were found to be involved in cellular senescence [32]. In the current study, the status of macrophage senescence was correlated to nuclear NF-*κ*B activity. In addition, the level of inflammatory cytokines including IL-6, TNF*α*, and IL-10 changed following the change in the status of macrophage senescence, suggesting a different secretory phenotype for such macrophages that were regulated by NF-*κ*B activity. Taken together, during PAO1-induced macrophage senescence, the NADPH oxidase activation induces ROS production, which partially regulates NF-*κ*B activation and its downstream cytokine release.

The production of ROS and NF-*κ*B-regulated inflammatory cytokines is essential for combating pathogens; however, the exaggerated elevation of these mediators may cause tissue damage and cell dysfunction. Herein, the capacity of phagocytosis in macrophage was decreased with elevated bacterial infection and overexpression of gp91^phox^ in macrophages, which abated their capacity in phagocytosis. Moreover, cellular senescence deteriorated under these circumstances, which might be attributed to the phagocytosis assay conducted in an infection model before the use of labeled beads. Also, after maximum phagocytosis, the phenomenon in macrophage might stabilize during severe bacterial infection. Furthermore, a previous report indicated that during PA infection, macrophage phagocytosis did not rely upon the activation of NF-*κ*B [33]. Taken together, the cellular senescence caused by PA infection in the macrophage might exhibit a decreased capacity of phagocytosis. This might partially explicate why severe infection with high bacteria load caused high mortality and morbidity. Although we did not test the role of macrophage senescence in an animal model with PA infection, Wen et al. utilized a PA lung infection model and demonstrated severe manifestations and lung injury in the aged rat than in the young rat. In their study, macrophage infiltration was postponed, and the number in the lung section was less in the aged rat than in the young rat after PA infection [34]. These data indicated that a cell growth arrest was probable in macrophages during PA lung infection in the aged rat. However, further animal experiments are essential for substantiating the effect of macrophage senescence on host defense during PA infection.

In conclusion, macrophages maneuver towards senescence under PAO1 infection. The interaction in the NADPH oxidase-ROS-NF-*κ*B pathway is involved in the process of macrophage senescence during PA infection. These senescent macrophages caused by high bacteria loads exhibit a compromised phagocytotic ability.
